# Nurses’ Self-Leadership in the Face of Challenging Situations, Such as Patient Deterioration

**DOI:** 10.1177/23779608241274232

**Published:** 2024-11-04

**Authors:** Carine Prinsloo

**Affiliations:** 1 Department of Health Studies, 56866 University of South Africa, Pretoria, Gauteng, South Africa

**Keywords:** nurses, nursing practice, patient deterioration, patient care, self-leadership

## Abstract

**Background:**

Nurses play an essential role as frontline caregivers, particularly in recognizing and responding to patient deterioration. This study addresses nurses’ self-leadership in the face of challenging situations such as patient deterioration, emphasizing the importance of self-leadership skills in navigating complex patient care scenarios. Delayed recognition of subtle changes in patient’s conditions has been linked to adverse outcomes, necessitating a closer examination of the role of self-leadership in nursing practice**.**

**Objective:**

The objective of this research was to explore and understand the implications of self-leadership among nurses when confronted with challenging situations, specifically focusing on patient deterioration.

**Methods:**

A qualitative, exploratory, and descriptive research design was employed, utilizing focus group discussions (FGDs) with nurses from a private hospital in Pretoria, South Africa. Semistructured interview guide facilitated the FGDs, and data saturation was achieved after the eight sessions. The sample included nurses with various qualifications and shifts, offering diverse perspectives on self-leadership in patient care. Rigorous data collection and trustworthiness measures, including member checking and verbatim transcriptions, were employed to enhance the conformability of the study.

**Results:**

The findings revealed six overarching themes and 15 subthemes, demonstrating the interconnectedness of self-leadership and nursing practice. Themes included responsibility and autonomy, compassion and care, support and collaboration, learning from experience, teamwork, and appreciation and recognition. These themes explained how nurses actively engaged in self-observation, constructive thought patterns, and natural reward strategies to proactively address patient deterioration.

**Conclusion:**

The study underlines the significance of integrating self-leadership principles into nursing practice. Embracing self-leadership in nursing holds promise for improving patient outcomes and nurturing a resilient and competent nursing workforce.

## Introduction

Timely recognition and response to patient deterioration are critical in healthcare for optimal outcomes. Nurses play a central role in this process, needing the skills to identify and act upon signs of deterioration promptly. Delayed detection can lead to prolonged hospital stays (Barwise et al., 2016), transfers to the intensive care unit (ICU) (Johnston et al., 2015), and an increased risk of mortality (Kang et al., 2016). Many patients deteriorate in general wards without timely recognition by nurses, often displaying abnormal vital signs for a significant period before intervention. To address this, hospitals globally utilize early warning systems and critical care outreach teams for improved detection and response to patient deterioration.

## Literature Review

Vincent et al. (2018) highlighted the increased challenge for nurses in general wards to detect changes in patient conditions. Factors contributing to this challenge include infrequent routine patient assessments, higher nurse-to-patient ratios, limited monitoring technology utilization, and the prevalence of complex comorbidities (Huber et al., 2021; Vincent et al., 2018). Nassief et al. (2021) indicate that factors contributing to instances of patient deterioration involve a lack of staff communication, errors in medical management, delayed treatment, inaccuracies in medical documentation, lapses in nursing management, and ambiguities in policies or guidelines.

Massey et al. (2017) emphasized the crucial role of patient assessment in aiding nurses to identify deterioration. This involves observing and monitoring vital signs closely. Research emphasizes the vital link between nursing assessments and adverse patient outcomes, including mortality (Beals et al., 2021). Early warning systems such as modified early warning scores (MEWS) help to promptly identify patient deterioration and guide decisions on escalating care. Ward nurses often seek peer or senior nurse support when responding to such situations. (Massey et al., 2014). Less-experienced nurses, with limited clinical knowledge, may struggle to recognize subtle changes in a patient's condition, often relying on more experienced colleagues for guidance. (Dresser et al., 2023). Continuous education and skills training are vital in enabling nurses to recognize and respond to patient deterioration (McDonnell et al., 2013).

Dresser et al. (2023) state that nurses have a sense of responsibility in recognizing and responding to patient deterioration. According to Sibandze and Scafide (2018), nurses’ professional values influence their decision-making and the quality of care they render to their patients.

Nurses play a critical role in patient care, often serving as the first line of defense when it comes to recognizing and responding to patient deterioration. In this high-stress environment, self-leadership among nurses becomes increasingly significant. The self-leadership theory describes a method through which individuals can self-motivate and guide their own actions by employing three specific strategies: behavior-focused strategies, constructive thought patterns, and natural reward strategies (Neck et al., 2020). This approach emphasizes the importance of personal influence over one's behavior, encouraging individuals to actively engage in self-directed techniques to foster productivity and positive outcomes.

Behavior-focused strategies empower individuals to self-manage their behavior and encompass techniques such as self-observation, self-goal setting, self-cueing, self-rewarding, and self-correcting feedback (Neck et al., 2020). For instance, a nurse who recognizes a knowledge gap in applying MEWS might set a goal to gather information about MEWS, thereby enhancing their competence in caring for deteriorating patients.

Constructive thought patterns involve reconfiguring one's perceptual processes to foster more positive thinking, including the abandonment of irrational or pessimistic self-talk in favor of more constructive and optimistic self-talk (Neck et al., 2020). Mental imagery, another aspect of constructive thought patterns, allows individuals to visualize the successful execution of a task before undertaking it. For instance, a nurse feeling anxious about performing nursing care interventions on a deteriorating patient might find relief by mentally picturing themselves carrying out these interventions competently under the guidance of a more experienced colleague.

Natural reward strategies focus on the inherently motivating aspects of a task and involve infusing more enjoyable aspects into it or shifting attention away from less enjoyable elements (Neck et al., 2020). This approach results in increased feelings of competence, self-control, and a sense of purpose. For instance, a nurse performing routine tasks like measuring a patient's vital signs may derive satisfaction from the significance of accurately assessing a patient's health status.

The purpose of this article is to explore and understand how nurses take on self-leadership roles when confronted with challenging situations, such as when patients experience deterioration in their health to fill the gap in our understanding of nursing practices.

## Methodology

### Research Design

A qualitative, exploratory, descriptive design was followed to understand nurses’ self-leadership role when confronted with challenging situations. The research employed a semistructured interview guide to facilitate FGDs. Data saturation was reached after the eight FGDs which lasted between 45 and 75 min each.

### Sample

The study was carried out at a level 2 private hospital located in Pretoria, South Africa. The hospital comprises of nine general wards, which include two medical, obstetrics, two surgical, orthopedic, oncology, and pediatric units, and a critical care outreach service. The accessible population for this study included 203 permanently employed nurses who worked both day and night shifts and who were directly engaged in delivering nursing care to patients. Participants were purposefully selected to aid the researcher in understanding the role of nurses’ self-leadership when confronted with deteriorating patients (Creswell & Creswell, 2023). Inclusion criteria involved all categories of nurses, including registered nurses, staff nurses, and auxiliary nurses working in general wards. Nurses working in theatres, the emergency department, and higher-level care units, such as high care and intensive care, were excluded from the sample as well as the nurse managers of the general ward.

### Data Collection

Following the acquisition of ethical clearance from the University's ethics committee (12/7/6), the private hospital group (UNIV-2013-007B), and the hospital management. The participants in the FGDs were selected to be homogeneous, meaning they shared similar characteristics (Gray, 2017). This method involved selecting participants based on their level of nursing qualification, as defined in the South Africa Nursing Act 33 of 2005 (South Africa 2005).

To approach potential participants while preserving their anonymity, unit managers were asked to extend invitations to possible participants to take part in the study. Comprehensive information about the research was shared both verbally and in writing, and all participants were requested to sign consent forms before participating in FGD. In addition, participants were made fully aware of their ethical rights, which included the option to withdraw from the study at any point without facing any adverse consequences.

To ensure the quality and neutrality of the FGDs, the researcher enlisted the expertise of an independent moderator. This decision was motivated by the fact that many of the participants had prior acquaintance with the researcher, and the aim was to prevent any potential discomfort that might arise if the researcher led the FGDs and to limit the potential responses from participants they perceived as socially desirable.

Before conducting the main FGDs, a preliminary focus group discussion, often referred to as a pilot, was organized with professional nurses. This allowed the moderator to fine-tune the wording of the questions and to ensure that the discussions would effectively address the research objectives. The feedback from the pilot focus group indicated that the questions were clear and easy to understand, and it provided insights into the anticipated duration of the discussions.

All FGDs were conducted in English in accordance with the hospital's communication policy. This choice was made to eliminate language barriers during the FGDs. Subsequently, one participant from each of the nine general wards, all possessing the same level of qualification, was invited to participate in an FGD. To ensure homogeneity, FGDs were held separately for participants from each qualification level, as each level of nursing qualification is associated with a distinct scope of practice.

To capture the experiences of both day and night shift nurses, FGDs were scheduled during both shifts. During these sessions, audio recordings with the permission of participants were made to facilitate data analysis, and the researcher took field notes while observing the participants.

### Trustworthiness

Credibility in this study was established through extensive engagement and persistent observation during FGDs. The researcher dedicated time to addressing any questions raised by participants. Throughout the observation process, the researcher systematically observed, listened, questioned, and documented participants’ behaviors, expressions, and interactions, considering the social setting, location, and context. To enhance dependability, FGDs were digitally recorded. Additionally, a pilot test was conducted with a focus group to ensure participants understood the questions and that these questions sparked appropriate discussions. A detailed journal documented the steps taken during both data gathering and analysis. Confirmability, which aims to ensure that the data accurately represents participants’ information and that interpretations reflect their voices rather than the researcher's biases, motivations, or perspectives, was a primary concern (Polit & Beck, 2020). Member checking was employed to validate the accuracy of themes, interpretations, and conclusions. Participants concurred with the moderator during member checking, thereby validating the trustworthiness of the results. Verbatim transcriptions of the FGDs were conducted, and an audit trail was maintained to confirm transcription accuracy. The moderator further bolstered confirmability by offering clarifications and paraphrasing during the discussions. These meticulous strategies collectively contribute to the overall rigor and reliability of the study's findings.

### Data Analysis

Following each FGD, the data were transcribed verbatim. To protect the anonymity of participants, any personally identifiable information was carefully removed. Participants from each FGD were allocated a number instead of using their names. Notably, the data from the pilot FGD were found to be valuable and were incorporated into the overall data analysis. The data analysis process adhered to the computer-assisted NCT (noticing things, collecting things, and thinking about things) analysis approach (Friese, 2019). This systematic method involves the following key steps:

- Observations: During the initial phase of data analysis, the researcher made observations while reading through the transcribed data and reviewing field notes. These observations were documented either by taking notes or by assigning preliminary codes to them.
- Collecting similarities: In the “collecting things” phase, the researcher conducted repeated readings of the collected data, with a focus on highlighting similarities among various data items.
- Coding: Identified items were given preliminary codes. In cases where an item did not neatly fit into a specific category, codes were either adjusted or renamed to accommodate the data effectively.
- Pattern recognition: The “thinking process” stage involves the careful consideration of the noted and coded items to identify patterns and relationships within the data. This process allowed for the creation of themes and subthemes based on these patterns and relationships (Friese, 2019).

## Findings

The collected data from the FGDs underwent analysis, leading to the identification of six overarching themes and 15 subthemes (table 1). The study drew inspiration from the definition of self-leadership, which posits self-leadership as the process of influencing oneself to establish the self-direction and self-motivation necessary for optimal performance (Neck et al., 2016).

**Table 1.** Themes and Subthemes.

Themes	Subtheme
Responsibility and autonomy	Taking charge of the situation when the patient is deteriorating
	Being knowledgeable and competent
	Reporting and recording abnormalities
The essence of nursing as a holistic calling	
Support and collaboration	Calling for help and advice
	Having a safety net
	MEWS as a cue
Learning from experience	Learning from patients
	Learning from peers
	Improving oneself
Teamwork	Involvement of colleagues
	Sharing the workload
Appreciation and recognition	Receiving recognition
	Lack of appreciation and recognition

### Theme 1: Responsibility and Autonomy

Within the context of self-leadership emphasized the critical role nurses play in patient care, particularly in terms of taking charge in situations where a patient's condition is deteriorating. The responses from the participants revealed a deep commitment to acting swiftly and competently in response to emergent patient needs. One participant captured this ethos by stating, “You act fast before anything could happen,” highlighting the proactive stance nurses adopt. This proactivity is further illustrated by another participant who explained, “So we know them better and you pick up some of the things and you report them,” demonstrating the importance of close patient observation and communication with the healthcare team.

### Subtheme: Being Knowledgeable and Competent

This subtheme underlined the foundational belief among participants that nurses must possess a comprehensive understanding of their patients’ conditions to provide the highest level of care. This belief is articulated by a nurse who responded, “To take charge and say, ok this is wrong with the patient, let's do something about it.” Another nurse emphasized the responsibility to be well informed about the patients under their care: “We are the ones who know the history of the patient, we know everything, we need to know risk factors, what is wrong with the patient, from the onset of admission.”

### Subtheme: Reporting and Recording Abnormalities

The diligent practices of nurses in monitoring patients and ensuring that any deviations from the norm are immediately communicated to higher authorities were captured. A participant described this process: “You do observations, and you find these abnormalities, you report to the sister [referring to the professional nurse].” Another adds, “If I see the patient is complicated and the condition is unstable, then I have to report to the sister,” highlighting the critical communication lines within patient care protocols.

### Theme 2: The Essence of Nursing as a Holistic Calling

Participants in the study expressed deep dedication to nursing, viewing it as a calling that demands doing what is right for patients. Nursing was described as integral to one's identity, bringing fulfillment and alignment with purpose. Motivated by a desire to bring happiness and comfort to patients, nurses expressed a commitment that transcended professional duty, rooted in love and service: “I am here to make the patient happy, to give them comfort.” The theme further explored the depth of nurses’ commitment to their patients, which illustrated a drive to provide care that goes beyond the professional obligation. They saw their role as akin to caring for family, with pride and fulfillment derived from saving lives. Nursing was portrayed not just as a job but as a profound commitment to patient well-being, integrating clinical excellence with compassionate care.: “It is caring, and love and to serve that patient, because when you are nursing a patient, it is like you think of, even if it is an adult person in our care, maybe this is can be my mother, so let me just nurse the patient as if this is my mother.”

### Theme 3: Support and Collaboration

The theme emerged as a critical component in nursing care, which captured the participants’ experiences and the significance of having reliable support and collaborative efforts, especially in critical patient care situations.

### Subtheme: Calling for Help and Advice

Participants emphasized the importance of seeking assistance and guidance from other healthcare professionals to manage patient health crises effectively: “And then they need more attention and then you call the outreach sister [referring to the critical care outreach team] to come and see the patient.”

### Subtheme: Having a Safety Net

Nurses find comfort in knowing there is backup available during emergencies. Statements like, “Normally if there is a crisis we can even call the code blue; they also come and help us,” and “Since we have the outreach sisters also, I see we are saving all the time.” This reassurance is captured in the participant's response, “Well for me it's to know that there is somebody that you can call.”

### Subtheme: MEWS as a Cue

The modified early warning score (MEWS) system was recognized as an important tool in guiding nurses’ actions in deteriorating situations. “We got the MEWS score chart, they have done the chart for our MEWS to see if the observations are like this you can call the outreach if like this you have to inform the doctors. So that chart helps us a lot,” illustrating its role as a standardized cue for action.

### Theme 4: Learning From Experience

This theme emphasized how nurses not only expand their knowledge and skills through hands-on experiences but also how this continual learning process significantly contributes to both personal satisfaction and the enhancement of patient care quality.

### Subtheme: Learning From Patients

Nurses identified their interactions with patients as essential learning moments, the nurses utilized patient data to inform their clinical decisions and improve care practices. This learning situation is captured in reflections such as, “Most of the time I have learned a lot from patients who are deteriorating,” and “After observations, you must plan, maybe that blood pressure is high, you must give something to lower it.”

### Subtheme: Learning From Peers

The exchange of knowledge and support among colleagues is another vital aspect of learning in the nursing environment. Nurses benefited from the collective wisdom and experiences of their peers, evidenced by statements like, “You call, ja when you feel like you can't pinpoint that is when you call for help,” and “I learned a lot from them, really I learned a lot.”

### Subtheme: Improving Oneself

Nurses’ dedication to self-improvement was evident in their enthusiastic pursuit of new knowledge and skills. “We do a lot of training. I mean we do, there is the sister that does blood-gas training,” and “If I don't know something, I google. If I don't know how something looks like, I google it.” These attitudes reflected a deep-seated commitment to personal and professional development, with the recognition that there is always room for growth, “They can see the potential in you willing to learn some more and they see that you are eager to know what's wrong and the way in which you solved that problem.”

### Theme 5: Teamwork

Teamwork is essential in nursing, for enhancing both the working dynamics among nurses and the quality of patient care.

### Subtheme: Sharing the Workload

Participants highlighted the essential role of involving colleagues in patient care, especially in critical situations. Involving staff in decisions such as adjusting oxygen levels demonstrated collaborative patient management: “You must involve the staff that is working with you. Now let's try to wean the oxygen, now let's put it down, now let's put it up, you are part of the thing before she (referring to the critical care outreach team) went to the sister.” This approach fosters a shared responsibility and collective engagement in the caregiving process.

### Theme 6: Appreciation and Recognition

Recognition and appreciation within the nursing field are crucial for motivating staff and acknowledging their hard work and achievements.

### Subtheme: Receiving Recognition

Nurses expressed how recognition from colleagues and superiors significantly boosted their morale and sense of accomplishment. Statements like “They say thank you very much for what you have done, you have done a good job, you have saved a life” and “They give us a certificate of appreciation” highlighted the positive impact that acknowledgment can have on nurses’ performance and well-being.

### Subtheme: Lack of Appreciation and Recognition

The absence of appreciation can be disheartening for nurses who dedicate themselves to their patients and their profession. One participant expressed her disheartened as follows, “By the time they come in the morning they don't appreciate, they don't. It is like you don't want a reward, you don't want a trophy, you just want a thank you for looking after this ward well done, that's all, but they don't.” This reflected a desire for acknowledgment and validation of their hard work and dedication.

## Discussion of Findings

The findings from the FGDs align closely with the principles of self-leadership which emphasize self-influence, self-motivation, and self-direction to achieve desired outcomes (Jooste & Roux, 2014). The data revealed a clear connection between self-leadership and the enhancement of personal effectiveness through three distinct approaches to individual-level actions, namely behavior-focused actions, natural reward (motivational) actions, and constructive thought (cognitive) patterns (Neck et al., 2020).

Each of these action approaches was prominently evident within the identified themes derived from the data in this study. This implies that the data not only supports but also aligns with the theoretical framework of self-leadership, showcasing its applicability in understanding and fostering personal effectiveness.

## Responsibility and Autonomy

Nurses exhibit a strong sense of responsibility and autonomy in their roles, particularly in situations where patient conditions are deteriorating. The ability to act swiftly and competently reflects a high degree of self-leadership among nurses, as they are required to lead themselves in performing tasks that are critical yet challenging. This aspect of nursing is supported by the concept of self-leadership, which involves leading oneself toward the performance of tasks through self-direction and self-motivation (Jooste & Roux, 2014). Participants’ responsibility to respond promptly to patients’ deterioration reflects a proactive approach where nurses motivate themselves to establish self-direction in patient care. According to Neck et al. (2016), self-observation is critical for recognizing the need for intervention and is a key component of self-leadership. This skill enables nurses to monitor their environment, assess patient conditions, and communicate effectively with the healthcare team, ensuring a timely response to any changes in patient conditions. The autonomy expressed by nurses in taking charge of situations reflects an empowered stance that is essential for effective self-leadership. Empowerment is linked to enhanced job satisfaction and increased motivation (Neck et al., 2020). Nurses through their autonomous practice and proactive approach embody the principles of self-leadership which impact the quality of patient care.

Being knowledgeable and competent is foundational to the nursing profession. This belief aligns with the self-leadership principle that individuals must possess a comprehensive understanding of their roles and responsibilities to perform effectively. Self-leadership encourages individuals to engage in self-reflection and self-assessment to enhance their personal and professional development, thereby ensuring they are competent and knowledgeable in their practice (Matahela & van Rensburg, 2023). Behavior-focused approaches involve, among other things, self-observation, self-goal setting, and self-cueing (Neck et al., 2016). Knowledge is a source of competence as it helps nurses to understand their patients’ conditions and the proposed outcomes for the patient. Focusing on the aspects of the task that increase their knowledge and competence, nurses can create a positive feedback loop that reinforces their self-motivation and satisfaction. This leads to a positive feeling of confidence and self-efficacy, which in turn enhances the performance of enjoyment of a task which can be seen as a natural reward approach to self-leadership (Neck et al., 2016).

The ability to recognize when situations deviate from the norm and to take appropriate action involves self-reflection, which enables nurses to understand their critical role when patients start to deteriorate. Nurses reflecting on their clinical practices can enhance their self-leadership skills, including the critical task of effectively reporting and recording patient care abnormalities (Matahela & van Rensburg, 2023). This portrays a picture of nursing as a profession deeply rooted in the principles of self-leadership, where taking responsibility, possessing a strong knowledge base, and effective communication are paramount in ensuring patient well-being. The emphasis on autonomy and responsibility reflects a keen awareness among nurses of their integral role in the healthcare system, demonstrating a commitment to excellence in patient care.

## The Essence of Nursing as a Holistic Calling

Participants exhibit compassion and care in their nursing practice, which relates to self-leadership skills. Compassion and care are essential components of nursing as they enhance the quality of care and the well-being of patients (Younas & Maddigan, 2019). Participants expressed their passion and dedication to nursing as well as their belief that nursing is not a job but a profound calling. This indicates that nurses find nursing to be self-rewarding, fulfilling, and aligned with their values and goals. By focusing on the meaning and significance of their work, they enhance their self-motivation and satisfaction. Natural reward approaches involve seeking and creating aspects of a task that are enjoyable, interesting, or meaningful (Neck et al., 2020). The notion of nursing as a calling emphasizes this self-motivation, where nurses view their role not just as a job but as a vital part of their identity and purpose in life. This sense of calling and dedication is paramount in self-leadership, as it drives nurses to take initiative, make independent decisions, and go above and beyond in providing care. Self-leadership in nursing involves not only the self-regulation of tasks and responsibilities but also a deeper, self-driven commitment to the holistic well-being of patients. It encompasses the ability of nurses to lead themselves in providing care that addresses not just the physical needs of patients but their psychological, social, and spiritual needs as well. This holistic approach reflects nurses' self-leadership where nurses’ actions are motivated by their internal values, empathy, and a profound sense of purpose.

## Support and Collaboration

Support and collaboration display how the autonomy of ward nurses is enhanced through effective teamwork and support systems. Support and collaboration are essential for effective nursing as they facilitate communication and teamwork among nurses. The participants shared their strategies and actions asking other healthcare professionals for guidance and assistance. This behavior of seeking help and advice from other healthcare workers links to the behavior-focused strategy, which involves self-awareness (Neck et al., 2020). Self-awareness helps the individual to identify their strengths and weaknesses, evaluate their progress, and adjust their behavior accordingly. The actions and strategies highlighted by participants, such as consulting shift leaders or specialized teams, illustrate a proactive approach to patient care. These actions demonstrate nurses’ self-awareness, which is a component of self-leadership, enabling them to identify when and how to seek assistance to optimize patient outcomes. The critical care outreach team, for instance, serves as an essential resource for nurses managing deteriorating patient situations. This collaboration between the ward nurses and the critical care outreach team not only facilitates timely interventions but also enriches the nurses’ professional development, which in turn boosts nurses' confidence. The nurses experience this as self-rewarding (Neck et al., 2020).

The notion of a ‘safety net’ and the psychological comfort it provides to nursing staff is a testament to the importance of supportive organizational cultures in fostering self-leadership. The participants explained how having the outreach sister or the code blue team to support and help them in an emergency makes them feel safe and secure. This indicates that they find support and collaboration to be rewarding, satisfying, and reassuring. The support from the outreach sister or code blue team also boosts nurses' natural reward approach by making challenging tasks more manageable and fulfilling. Natural reward approaches involve seeking aspects of managing deteriorating patients that are enjoyable or meaningful (Neck et al., 2016).

The modified early warning score (MEWS) system is highlighted as an invaluable tool, which guides nurses in their decision-making process. This tool's standardization and objectivity support self-leadership by providing clear cues for action, thereby reinforcing safety and efficacy in patient management. The participants reported how they used the MEWS chart to monitor the patient's status, to decide when to call for help, and to ensure the safety of themselves and the patient. This implies that they use the MEWS as a cue to shape their cognitive processes and influence their behavior in the management of a patient with abnormal vital data. Behavior-focused approaches enable people to take control of their own behavior and include methods like self-cueing (Neck et al., 2020). The MEWS system demonstrates how structured tools and protocols can empower nurses to act confidently and autonomously, aligning with self-leadership principles.

## Learning From Experience

Self-leadership emphasizes the importance of self-influence, self-motivation, and self-direction to achieve personal and professional goals. The reflections shared by nurses on learning from patient care experiences and interactions underline a proactive approach to knowledge acquisition and application. Nurses indicated that they learn from experience in their nursing practice, which is essential for nursing as it helps nurses develop clinical competence, professional judgment, and reflective practices. The participants acknowledge that patients' baseline observations are a valuable source of information and feedback, and they use the patients’ essential information to motivate their decisions and behaviors. This implies that nurses use the following behavior-focused approach namely self-awareness to monitor their performance, evaluate their outcomes, and adjust their behavior accordingly. Self-awareness helps nurses to identify their strengths and weaknesses, improve their skills, and overcome challenges. Self-awareness also helps individuals to set realistic and challenging goals for themselves, which is another aspect of the behavior-focused strategy. Self-goal setting is the process of defining what one wants to achieve and how to achieve it. Self-goal setting helps the individual to focus their attention, direct their efforts, and increase their persistence (Neck et al., 2020).

The participants describe the exchange of information and advice with their colleagues and how they benefit from the support and validation of their peers. This indicates that they find learning from peers to be rewarding, satisfying, and motivating. By focusing on the social aspects of their work environment, they enhance their self-motivation and satisfaction which involves a natural reward approach. Engaging in self-reward strategies that acknowledge and value the contribution and support of peers can lead to a more motivated work environment (Inam et al., 2023).

Nurses’ dedication to self-improvement, through engaging in training programs and self-initiated research, exemplifies the self-leadership principle of self-goal setting and constructive thought patterns. The participants’ proactive approach to gaining new knowledge and skills, which is reflected in their desire to seize opportunities for training and personal development, demonstrates their positive attitude toward learning. This implies that they use constructive thought processes such as positive self-talk, mental imagery, and beliefs and assumptions to shape their cognitive processes and influence their beliefs and attitudes. Constructive thought pattern approaches can be observed as aiding nurses in enhancing their self-confidence, self-efficacy, and self-esteem (Stringer, 2021).

## Teamwork

Teamwork and self-leadership are interconnected concepts that complement each other. A strong sense of self-leadership within each team member could enhance overall teamwork leading to a more productive effort. Participants expressed satisfaction in teamwork when fellow nurses assisted them in managing nursing care for their patients and when actively participating in addressing a patient's condition. Nurses’ self-satisfaction derived from collaboration and contributing to the nursing care of deteriorating patients contributes to a positive and fulfilling work environment. This can be viewed as a natural reward approach for nurses (Neck et al., 2020).

## Appreciation and Recognition

The positive feedback loop created by recognition from colleagues and superiors can reinforce nurses’ self-leadership behaviors. Specifically, appreciation acts as an external self-reward that can enhance intrinsic motivation, leading to a higher engagement in self-directed behavior and personal excellence. Recognition validates the nurses’ efforts and aligns with the self-leadership strategy of focusing on natural rewards, where individuals seek out and emphasize the inherently rewarding aspects of their work. The statements from nurses highlight how recognition significantly contributes to their sense of pride and accomplishment, which is crucial for sustaining motivation and commitment to high-quality patient care. This aligns with research by Alahiane et al. (2023), who found that recognition positively affects employees’ self-efficacy, intrinsic motivation, and ultimately, their job performance. On the other hand, conversely, the absence of appreciation can lead to feelings of undervaluation and disheartenment, which might undermine the self-leadership strategies nurses employ to maintain their motivation and performance.

## Strengths and Limitations

The qualitative research design used a diverse sample that included registered nurses, staff nurses, and auxiliary nurses which provided a comprehensive perspective on the self-leadership of nurses across the different levels of nursing practice, and the inclusion of day and night shift nurses ensured a diverse range of experiences.

The research conducted in a single private hospital limits the generalizability of the findings to other healthcare settings.

## Implications of Nursing Practice

The findings have implications for nursing practice, especially in the context of recognizing and responding to patient deterioration. Understanding how ward nurses exercise self-leadership in challenging situations can lead to improved patient care and safety. Identifying effective self-leadership strategies can help, enhance patient monitoring and ultimately save lives. This research can assist in the development of training and educational programs that empower nurses to become better self-leaders in challenging scenarios. Although competence was not singled out as a challenge, through the application of self-leadership, nurses recognize their lack of knowledge and skills and are motivated to enhance these areas, aiming to achieve greater competency in patient care.

## Conclusion

This research examines the field of nursing practice, casting insight into the role of self-leadership in the context of recognizing and responding to patient deterioration. The findings highlight the multifaceted nature of self-leadership, involving behavior-focused strategies, constructive thought patterns, and natural reward strategies, all of which are crucial for nurses facing the challenges inherent to patient care.
